# Patients’ assessments of the continuity of primary care in Finland: a 15-year follow-up questionnaire survey

**DOI:** 10.3399/bjgp14X681841

**Published:** 2014-09-29

**Authors:** Risto Raivio, Doris Holmberg-Marttila, Kari J Mattila

**Affiliations:** Joint Authority of Päijät-Häme Social and Health Care Group, Lahti, Finland.; Pirkanmaa Hospital District, Tampere, Finland.; University of Tampere, Tampere, Finland.

**Keywords:** continuity of care, follow-up study, general practice, primary health care, quality of care, questionnaire

## Abstract

**Background:**

Continuity of care is an essential aspect of quality in general practice. This study is the first systematic follow-up of Finnish primary care patients’ assessments with regard to personal continuity of care.

**Aim:**

To ascertain whether patient-reported longitudinal personal continuity of care is related to patient characteristics and their consultation experiences, and how this had changed over the study period.

**Design and setting:**

A 15-year follow-up questionnaire survey that took place at Tampere University Hospital catchment area, Finland.

**Method:**

The survey was conducted among patients attending health centres in the Tampere University Hospital catchment area from 1998 until 2013. From a sample of 363 464 patients, a total of 157 549 responded. The responses of patients who had visited a doctor during the survey weeks (*n* = 97 468) were analysed. Continuity of care was assessed by asking the question: ‘When visiting the health centre, do you usually see the same doctor?’; patients could answer ‘yes’ or ‘no’.

**Results:**

Approximately half of the responders had met the same doctor when visiting the healthcare centre. Personal continuity of care decreased by 15 percentage points (from 66% to 51%) during the study years. The sense of continuity was linked to several patients’ experiences of the consultation. The most prominent factor contributing to the sense of continuity of care was having a doctor who was specifically appointed (odds ratio 7.28, 95% confidence interval = 6.65 to 7.96).

**Conclusion:**

Continuity of care was proven to enhance the experienced quality of primary care. Patients felt that continuity of care was best realised when they could consult a doctor who had been specifically appointed to them. Despite efforts of the authorities, over the past 15 years patient-reported continuity of care has declined in Finland.

## INTRODUCTION

Continuity of care is an essential aspect of good-quality primary care, as is involving patients in assessing, developing, and improving it.^1^–^6^ The primary care profession must constantly aim to maintain this. Various attempts have been made to establish a consensus on continuity of practice as a basis for valid and reliable assessment of primary care in different healthcare settings and dimensions.^4^–^9^ Continuity of care can be assessed by the general population, the users (patients), or the providers (professionals or organisations), and is related to other healthcare dimensions and outcomes.

Continuity of care constitutes an indicator of quality in general practice.^8^ There are a number of studies and articles on the criteria for indicators and instruments used in assessing continuity of care, including literature focusing on measurement of patients’ views of primary care.^4^,^7^–^14^ Patients place great value on the ability to see the same doctor;^15^ Hjortdahl and Laerum^16^ showed that the personal continuity of care is positively linked to patients’ overall satisfaction. Other studies have, likewise, found strong evidence of the correlation between continuity of care and improved patient satisfaction.^7^,^17^,^18^

A review by Starfield *et al* 19 emphasises that continuity of care is one core dimension in a robust primary care system; they also found continuity of care to be cost effective and promotive of greater efficiency of services. Kringos *et al* 8 have assessed the relevance of continuity of primary care in relation to other primary care dimensions and healthcare system outcomes; in their review of the literature, they found associations with coordination, comprehensiveness, quality, efficiency, population health, patient satisfaction, costs, and strength of primary care.

For the GP, continuity of care:

- strengthens the doctor–patient relationship and the sense of partnership in care;
- improves diagnostic and communication skills;
- enhances trust and empathy;^4^,^5^ and
- improves the coordination and integration of care.^12^

According to the European Definition of General Practice/Family Medicine, a GP is responsible for the provision of longitudinal continuity of care, as determined by the needs of the patient.^20^ Moreover, the significance of continuity challenges GPs to develop teamwork with other professionals in their practices to engage them in promoting and improving the health of their patients.^21^

Patients are increasingly perceived as clients in healthcare services and experts in their own care and, as such, GPs must make additional efforts to recognise patients who are chronically ill as partners in care.^22^ Both GPs and their patients seem to realise the value of maintaining a good doctor–patient relationship. Furthermore, patients describe consistent relationships in primary care as a reassuring, positive, and secure partnership.^23^

**How this fits in**

There is extensive literature on the benefits of continuity of care for patients, professionals, authorities, and healthcare systems. The personal continuity of consultations in primary care in Finland is decreasing; this is a matter of concern. This study shows that having a specific named doctor generates continuity and patients experience better quality health care as a result.

Despite many positive findings in this dimension of general practice, there is ongoing debate as to whether, and to whom, the continuity of care really matters.^24^–^26^ There is variation between different patient groups and primary healthcare organisations regarding the level of satisfaction with continuity of care.^12^–^17^, ^26^–^30^ Patients using primary care services are generally fairly satisfied in this respect.

Finnish healthcare services offer universal coverage for a comprehensive range of care, which is delivered primarily by organisations that are publicly owned and operated;^31^ primary healthcare services are provided mainly by municipal health centres that are publicly funded. The Finnish primary healthcare system is health centre-oriented and wide, both in terms of the numbers of staff and the different professions employed.^32^–^34^ The actual size and population of primary care health centres varies considerably but staff work mostly in pairs or teams, and the distribution of tasks from doctors to nurses is common. Practice nurses who assess the need and urgency of care are almost invariably patients’ first contacts, either on the telephone or face to face.

Only about half of health centres have a personal list system, with all patients allocated to a named family doctor. In addition, free occupational health services also play an important role in Finnish health care by providing primary care to the employed population. In Finland, patients can choose to consult a doctor either in a health centre or in occupational health. They are also able to choose a private doctor, for which they will have to pay extra. In the past 10 years, the authorities in Finland have sought to improve the quality of primary health care, increase its resources and integration of care, and emphasise patients’ roles in their own care.^32^–^34^

To the authors’ knowledge, prior to this investigation, there have been no systematic longitudinal studies measuring continuity of care in Finland. The aim of this study was to ascertain:

- how personal, longitudinal continuity of care is related to certain patient characteristics; and
- what were patients’ experiences of consultations with doctors working in Finnish primary care centres.

How the patient-reported, personal, continuity of health-centre doctors had changed over the study period was also examined.

## METHOD

### Design

The Department of General Practice at the University of Tampere sent a questionnaire (available from the authors on request) to 65 primary healthcare centres in 1998, 1999, 2000, 2001, 2003, 2005, 2007, 2009, 2011, and 2013. In every study year, the questionnaire was given to patients attending for treatment during one particular week. The questions were based on international studies^34^ and adapted to the special characteristics of Finnish primary health care.^35^

The questionnaire was piloted in the Pirkanmaa area in 1998, given to 19 399 patients and 9276 patients responded (a 48% response rate). In 1999, the study was extended to primary healthcare centres located in the catchment area of Tampere University Hospital. There were 65 health centres in this area, serving a total population of 1.2 million.

Data were collected during week 39, in September. Reception staff distributed the questionnaire to patients visiting physicians and nurses due to illness from Monday to Friday between 8am and 4pm. Patients placed the anonymously filled questionnaires in a box in the waiting room after their consultation. During the data-collection periods, 363 464 patients visited the practices, and 157 549 responded. The response rate varied yearly from 39% to 53%. The answers of patients (*n* = 97 468) who had visited a doctor during the survey weeks were then analysed.

Patients were asked for their background information, including:

- sex;
- age;
- reason for visit (acute event, other urgent matter, or non-urgent matter);
- visits prior to the present one;
- which healthcare provider the patient had met;
- evaluation of the consultation; and
- opinion of the visit.

From 2005, the question ‘Do you have a particular doctor appointed for you at the health centre?’ was added to the questionnaire. Responses in the affirmative signified that patients were allocated to a specific doctor by health organisations based on where they lived, without them having the freedom to choose which doctor they would see. From 2009 another question was added: ‘In the last 12 months, how many times have you visited the health centre prior to this visit?’.

The longitudinal, personal, continuity of care was assessed with the question: ‘When visiting the health centre, do you usually see the same doctor?’; possible responses were ‘yes’ and ‘no’. The responders graded the quality of service using a scale that is traditional in the Finnish school system and familiar to all patients; grades range from 4 (‘very poor’) to 10 (‘excellent’). The highest score, 10, was considered the best and, therefore, represented the best possible quality. The frequency of this top score was determined for each domain compared with other scores (4–9).

### Analyses

SPSS (version 20.0) was used for statistical analysis. The statistical significance of differences in frequencies between the groups was tested by χ^2^ test. Binary logistic regression analyses were used on patient characteristics, and on patients’ evaluation of consultation with their family doctor. To study how the various factors were connected to the continuity of care (dependent variable), both univariate and multivariate regression analyses were used. Patient-related factors and quality factors were dichotomised.

## RESULTS

The number of responders varied between questions and study years. All the responders did not answer all questions. The replies of those patients who visited a doctor during the study week for all study years were analysed. The total number of patients was 97 468 (Table 1).

**Table 1.** Responders who visited a doctor in health centres, by study year

**Year**	**Patients, *n***
1998	6377
1999	17 132
2000	14 887
2001	10 724
2003	9783
2005	10 540
2007	10 557
2009	5956
2011	5791
2013	5721

Total	97 468

Of patients who had seen a doctor, 64% were female, 45% were aged ≥60 years, 57% needed urgent or less urgent treatment, and 70% had visited the health centre at least once in the preceding 12 months. Of the responders, 66% reported having a specific appointed doctor at the health centre.

Table 2 details the responses relating to sex, age, urgency, whether the patient had visited the healthcare centre in the previous 12 months and, for those patients who were usually able to see the same doctor, whether the patient had an appointed doctor. Among patients who had a specific doctor appointed for them by the health centre, continuity of care was considered to mean the same thing in both male and female patients. In those aged ≥60 years, continuity of care was confirmed 11 percentage points more often than in the younger group. In non-urgent visits and in visits over the 12 months prior to the study visit, continuity of care was actualised slightly more often (more often than visits under 12 months). Of patients who said that they had a specific doctor appointed to them, 73% could usually meet the same doctor; only 27% usually met the same doctor if they had no appointed doctor (Table 2).

**Table 2.** Number of patients who were usually able to see the same doctor, by sex, age, urgency of consultation, prior visit, and specific appointed doctor

**Patient characteristics**	**Responders, *n***	**Do you usually meet the same doctor?**		
		**Yes,%**	**No,%**	***P*-value**
**Sex**				0.118
Female	58 934	62.8	37.2	
Male	32 911	62.3	37.7	

**Age**				≤0.001
<60 years	51 908	58.4	41.6	
≥60 years	33 009	69.6	30.4	

**Urgency**				≤0.001
Acute/less acute	49 404	58.7	41.3	
Non-urgent	36 657	67.1	32.9	

**Visit in previous 12 months^a^**				≤0.001
Yes	11 701	49.4	50.6	
No	5019	55.2	44.8	

**Specific doctor^b^**				≤0.001
Appointed	23 354	72.8	27.2	
Not appointed	11 915	26.6	73.4	

a Question added to survey in 2009.

b Question added to survey in 2005.

Overall, two-thirds of the patients gave the highest marks for quality aspects when they had a specific doctor appointed to them by the health centre (Table 3). The difference in giving the best-possible grades for the consultation between those who had a specific doctor appointed to them and those who did not varied from 7 to 10 percentage points and was statistically significant (*P*<0.001) in all quality aspects (Table 3).

**Table 3.** Consultation evaluation by patients who gave the highest score (10 points) for quality aspects of consultation when asked: ‘When you visit the health centre, do you usually see the same doctor?’

	**Responders, *n***	**Do you usually see the same doctor?**		
		**Yes, %**	**No, %**	***P*-value**
**Did you get information about the treatment options for your particular health problem?**				<0.001
Highest score	29 215	68.5	31.5	
Other	44 876	59.9	40.1	

**Did you get clear and adequate instructions for further care and treatment?**				<0.001
Highest score	35 322	67.7	32.3	
Other	42 459	59.4	40.6	

**Did the doctor/nurse listen to your problems and did they show interest toward you and willingness to answer your questions?**				<0.001
Highest score	40 598	67.5	32.5	
Other	42 197	58.3	41.7	

**Did you feel that your matters were dealt with confidentially?**				<0.001
Highest score	48 613	66.2	33.8	
Other	35 131	58.6	41.4	

**Did you get help for your health problem?**				<0.001
Highest score	37 930	66.4	33.6	
Other	39 294	59.9	40.1	

In both univariate and multivariate regression analyses, patient-related items explained the continuity of care; patients’ age, reason (urgent, non-urgent) for visit, and previous visits to the health centre (within the preceding 12 months) were linked to continuity of care (Table 4).

**Table 4.** Patient-related and consultation-related covariates representing continuity of care^a^ in univariate and multivariate regression analyses

	**Univariate analysis**		**Multivariate analysis^b^**	

	**OR (95% CI)**	***P*-value**	**OR (95% CI)**	***P*-value**
**Patient-related items**				
Woman	1.02 (0.99 to 1.05)	0.118	0.89 (0.82 to 0.98)	0.014
Aged ≥60 years	1.63 (1.58 to 1.68)	<0.001	1.45 (1.32 to 1.59)	<0.001
Non-urgent visit	1.43 (1.39 to 1.47)	<0.001	1.32 (1.21 to 1.44)	<0.001
Visit in preceding 12 months	1.26 (1.18 to 1.35)	<0.001	1.11 (1.01 to 1.22)	0.039
Appointed doctor	7.38 (7.02 to 7.75)	<0.001	7.28 (6.65 to 7.96)	<0.001

**Consultation evaluation**				
Got enough information	1.46 (1.41 to 1.51)	<0.001	1.03 (0.88 to 1.20)	0.743
Got adequate instructions	1.43 (1.39 to 1.48)	<0.001	1.02 (0.86 to 1.21)	0.812
Felt doctor listened and showed interest in their problems	1.49 (1.45 to 1.53)	<0.001	1.06 (1.92 to 1.23)	0.420
Felt confident about confidentiality	1.38 (1.34 to 1.42)	<0.001	1.21 (1.06 to 1.38)	0.006
Got help for their health problem	1.32 (1.29 to 1.36)	<0.001	1.10 (0.97 to 1.25)	0.135

a Continuity of care was determined by asking: ‘When visiting the health centre, do you usually see the same doctor?’

b All variables in the model. OR = odds ratio.

In the univariate analysis, patients’ experiences of how the doctor had listened and was willing to give answers were related to the continuity of care. In addition, the doctor’s behaviour during the consultation, the information given regarding medication and tests, confidentiality, the sense of receiving help with their problem, and receiving adequate instructions on further care contributed to the correlation (Table 4). In the multivariate analysis only the information and instructions given in the consultation did not appear to be significantly correlated with the continuity. The most prominent factor related to continuity of care was having a specific doctor appointed by the health centre for the consultation (odds ratio = 7.28, 95% confidence interval = 6.65 to 7.96).

**Figure 1.**
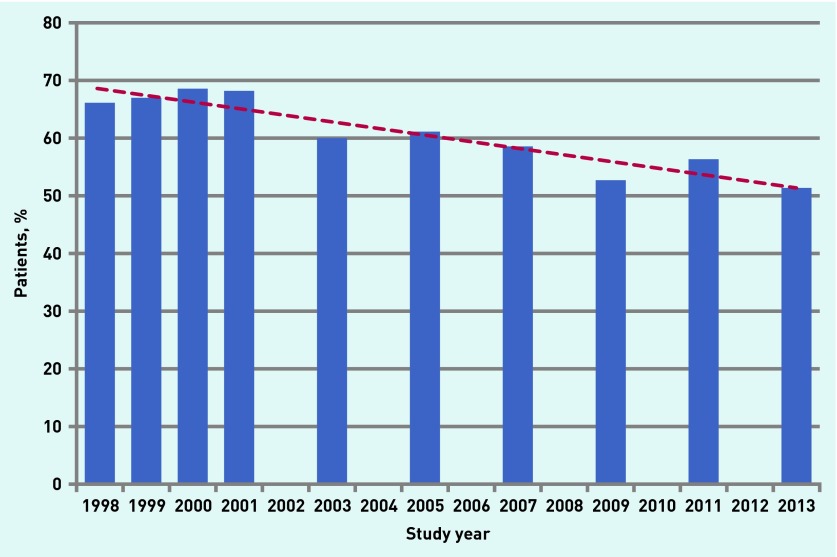
***The percentage and trend line of patients who experienced continuity of care because they usually saw the same doctor during the study period . Continuity of care was determined by asking: ‘When visiting the health centre, do you usually see the same doctor?’***

Personal continuity of care decreased by 15 percentage points (from 66% to 51%) from 1998 to 2013 (Figure 1). At most, over two-thirds (69%) of patients, in 2000, reported that when they visited the health centre, they usually saw the same doctor in that year; in 2013, only around half (51%) were of the same opinion.

## DISCUSSION

### Summary

Several patient characteristics and features of their consultation experiences proved to be connected to continuity of care. The most clearly determining factor was having a specific doctor appointed by the health centre. Also notable was patients’ experience of the confidentiality of their consultation and how the doctor listened and was willing to give answers. Older patients, in particular, seemed to value having a specific doctor appointed to them, who was able to listen, understand, and care.

During the study years, 1998–2013, the continuity of Finnish primary care did not improve.

### Strengths and limitations

This study constitutes the first longitudinal, systematic inquiry into continuity of primary health care in Finland. During the 15-year study period, it was possible to gather an extensive sample of patient opinions on personal continuity of care of health centre doctors. In this study area of 1.2 million inhabitants, there are both small, rural health centres and large health centres in the conurbations. However, the patients who chose to answer the questionnaire were likely to be those who had an opinion and wanted to express it. The responders’ views cannot be taken as representative of the whole population, therefore, but are likely to be representative of the main population using primary health care services in Finland.

The number of responders varied greatly during the study years. One of the reasons for this could be organisational and structural changes within, and between, the municipalities arranging primary healthcare services. This may also have been the result of the strengthening of occupational health services. Furthermore, some of the health centres suffered from a lack of professional healthcare staff. Although there were fewer responders, the number of patients visiting health centres also declined. Patients with relatively simple healthcare problems have gradually moved from health centres to occupational health services, while patients with long-term conditions and multimorbidity seem to remain under the responsibility of health centres. These patients also tend to need more services and longer appointments. At the same time it has become harder for people to access primary healthcare centres as resources are stretched.

Attempts have been made to find out reasons for non-response. Some examples are patients having left spectacles at home or being in a hurry; however, patients may not have completed the questionnaire because they had no desire or inclination to do so.

The researchers were aware of the challenges of studies using a questionnaire survey.^35^,^36^ Asking reception staff to pass out questionnaires is flawed as a means of systematic inquiry and involves a notable sampling bias. As the same flaws apply to the data throughout, however, it is reasonable to conclude that comparison over time is still robust. The low overall response rate (45%) is a limitation; nonetheless, the researchers consider the data and the process of assessing patients’ views to be reliable and comprehensive.^37^ The adoption of the highest score was inspired by its use in other patient-satisfaction measurements.^38^

### Comparison with existing literature

The results of this study show that patients were less satisfied with the continuity of care at the end of the study, in 2013, than they were at the outset. A number of factors are associated with continuity of care,^14^–^17^ some of which are related to patient characteristics and some to features of the healthcare system. Several studies have shown that patients’ age, sex, perceived health status at the time, and socioeconomic status have an effect on continuity of care.^27^–^30^ The variables mentioned did not change significantly in Finland over the course of the study period and those associated with continuity of care here were similar to those in previous studies that used a similar methodology.^39^,^40^

Patients in primary care appreciate continuity;^15^–^17^ on the other hand, continuity of care alone is not a guarantee of good and efficient health care. The clearest determining factor associated with continuity of care in this study was having a specific doctor appointed by the health centre. Older patients with chronic diseases seem to benefit from the continuity of care and a long treatment relationship with the same doctor and nurse;^38^ in this study, patient age was related to the continuity of care as much as the non-urgency of the visit or previous visits to the doctor.

Good communication, proper instructions, and confidentiality during the consultation have been shown to increase satisfaction and enhance the continuity of care.^40^ These aspects were also found in this study. When trust and communication between patient and doctor is good enough, the patient tends to be satisfied with, and also committed to, their care.^40^

There has been some discussion both internationally and also in Finland about whether personal list systems with patients being allocated to a specific named doctor are desirable, and feasible, in a primary care setting. A specific named doctor seems to lead to fewer critical and uninformed patients.^4^,^5^,^26^,^34^,^40^ The new Finnish Health Care Act, valid from 2010,^41^ emphasises freedom of choice for all patients, with authorities now appearing to trust that patients are both able, as well as willing, to choose for themselves the doctor with whom they prefer to consult. The question becomes, then, whether authorities are able to put this choice into practice when financial resources in health care are decreasing.

### Implications for practice

The declined continuity of care in Finland is far from desirable and it would seem that the number of patients who could most profit from having a specific appointed doctor, that is, from being able to see the same physician, has increased. It is disconcerting that continuity of care has not improved in line with this. Patients who would particularly benefit from having an appointed doctor include those who are fragile, such as older patients with long-term conditions and multimorbidity, and patients who need, or use, the care most, such as drug and alcohol misusers, those who are mentally ill, and young people and families with social problems. These patients could profit from having a care manager, who, together with a team of professionals, would not only integrate and coordinate their care, but also empower patients to take an interest in their own care. The possibility of choosing their own doctor would be ideal for these patients but having a specific family doctor assigned to them would, at least, ensure the continuity of their care.

This study underlines the importance of the patient in assessing primary care services. It also confirms the significance of having a doctor personally assigned and how aspects of care that indicate good quality care can also promote continuity. The findings also indicate that new means of coordinating and developing care in Finland are still necessary to improve the continuity of primary health care. The authors suggest that, at least, those patients who need care most should be able to consult with a specific family doctor to enhance the continuity and quality of their care.
